# The midgut transcriptome of *Phlebotomus *(*Larroussius*) *perniciosus*, a vector of *Leishmania infantum*: comparison of sugar fed and blood fed sand flies

**DOI:** 10.1186/1471-2164-12-223

**Published:** 2011-05-10

**Authors:** Anna Dostálová, Jan Votýpka, Amanda J Favreau, Kent D Barbian, Petr Volf, Jesus G Valenzuela, Ryan C Jochim

**Affiliations:** 1 Department of Parasitology, Faculty of Science, Charles University in Prague, Vinicna 7, 128 44 Praha 2, Czech Republic; 2 Genomics Unit, Research Technologies Section, Rocky Mountain Laboratories, Hamilton, Montana 59840, USA; 3 Vector Molecular Biology Unit, Laboratory of Malaria and Vector Research, NIAID, NIH, Rockville, MD, 20852, USA

## Abstract

**Background:**

Parasite-vector interactions are fundamental in the transmission of vector-borne diseases such as leishmaniasis. *Leishmania *development in the vector sand fly is confined to the digestive tract, where sand fly midgut molecules interact with the parasites. In this work we sequenced and analyzed two midgut-specific cDNA libraries from sugar fed and blood fed female *Phlebotomus perniciosus *and compared the transcript expression profiles.

**Results:**

A total of 4111 high quality sequences were obtained from the two libraries and assembled into 370 contigs and 1085 singletons. Molecules with putative roles in blood meal digestion, peritrophic matrix formation, immunity and response to oxidative stress were identified, including proteins that were not previously reported in sand flies. These molecules were evaluated relative to other published sand fly transcripts. Comparative analysis of the two libraries revealed transcripts differentially expressed in response to blood feeding. Molecules up regulated by blood feeding include a putative peritrophin (*PperPer1*), two chymotrypsin-like proteins (*PperChym1 *and *PperChym2*), a putative trypsin (*PperTryp3*) and four putative microvillar proteins (*PperMVP1*, *2*, *4 *and *5*). Additionally, several transcripts were more abundant in the sugar fed midgut, such as two putative trypsins (*PperTryp1 *and *PperTryp2*), a chymotrypsin (*PperChym3*) and a microvillar protein (*PperMVP3*). We performed a detailed temporal expression profile analysis of the putative trypsin transcripts using qPCR and confirmed the expression of blood-induced and blood-repressed trypsins. Trypsin expression was measured in *Leishmania infantum*-infected and uninfected sand flies, which identified the *L. infantum*-induced down regulation of *PperTryp3 *at 24 hours post-blood meal.

**Conclusion:**

This midgut tissue-specific transcriptome provides insight into the molecules expressed in the midgut of *P. perniciosus*, an important vector of visceral leishmaniasis in the Old World. Through the comparative analysis of the libraries we identified molecules differentially expressed during blood meal digestion. Additionally, this study provides a detailed comparison to transcripts of other sand flies. Moreover, our analysis of putative trypsins demonstrated that *L. infantum *infection can reduce the transcript abundance of trypsin *PperTryp3 *in the midgut of *P. perniciosus*.

## Background

Leishmaniases are a group of vector-borne diseases caused by parasitic protozoa of the genus *Leishmania. Leishmania infantum *(syn. *L. chagasi*) is the main etiological agent of zoonotic visceral leishmaniasis, the most deadly form of the disease. The lack of a human vaccine, increasing resistance to the currently used drugs and their serious side effects urge the need for research of visceral leishmaniasis. In the western and central part of the Mediterranean basin, the major vector of *L. infantum *is *Phlebotomus perniciosus *[1].

*Leishmania *amastigotes are ingested with the blood meal upon female sand fly feeding on the mammalian host. After a series of morphological changes, propagation and migration of the parasites to the anterior part of the midgut, the infection is transmitted to another host during the next blood feeding. In hematophagous arthropods, blood feeding induces a number of processes including digestion, metabolism, diuresis and egg development. Unlike many other arthropod-borne infections, e.g., *Plasmodium *in mosquitoes, *Leishmania *complete their whole developmental cycle within the midgut of the sand fly. Several natural barriers to *Leishmania *development in the midgut have been described including the secreted proteolytic enzymes, the peritrophic matrix surrounding the ingested blood meal and the necessity to bind to the midgut epithelium (reviewed by [2]). Thus, the midgut is the primary organ where interactions between the vector sand fly and the parasite occur and it represents a key target for interruption of *Leishmania *transmission.

While the genome sequences of several *Leishmania *species, including *L. infantum*, have been published [3] and molecular studies abound, molecular data on sand flies are limited. An analysis of expressed sequence tags (ESTs) from the whole *Lutzomyia longipalpis *sand fly and salivary gland transcriptomes of several sand fly species have been published (reviewed by [4]). With regard to *Leishmania *development in the midgut, particularly midgut-specific transcriptomic analyses, studies of *L. longipalpis *and *Phlebotomus papatasi *[5-7] have brought important insights into the repertoire of molecules expressed in the midgut. Several midgut proteins from these two species were functionally characterized [8] and shown to impact *Leishmania *development [9,10].

In this study, we have generated and sequenced two cDNA libraries from the midgut tissue of *P. perniciosus *and analysed sequences present both before and after blood feeding. Furthermore, we provide phylogenetic analysis and comparison with the midgut molecules described in *L. longipalpis *and *P. papatasi*. Comparison of these three species is especially valuable with regard to *Leishmania *transmission. *P. papatasi *is the principal vector of cutaneous leishmaniasis caused by *Leishmania major *in the Old World [1]. It is refractory to the development of other species of *Leishmania *[11]. *Lutzomyia longipalpis *is the vector of *L. infantum *(*chagasi*) in Latin America and is considered a permissive vector due to full development of various *Leishmania *species in laboratory infections [1,12]. While being phylogenetically closer to *P. papatasi*, in some aspects *P. perniciosus *resembles *L. longipalpis*. First, it is a natural vector of *L. infantum*. Second, it is also permissive to the development of other *Leishmania *species [13]. Therefore, the present study provides a valuable database for identification of vector molecules that affect the vectorial competence of sand flies.

## Results and Discussion

**Figure 1:**
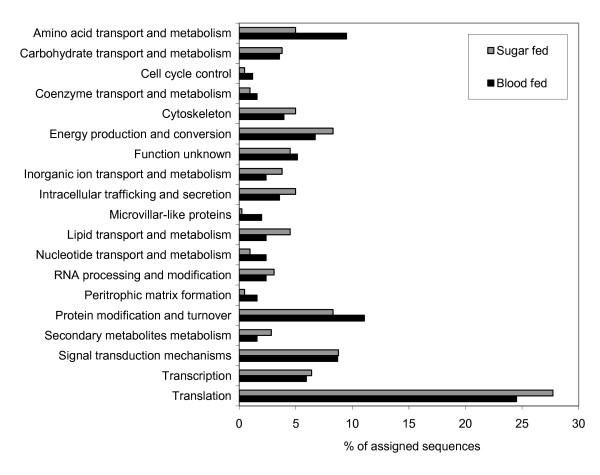
**Distribution of clusters from the sugar fed and blood fed libraries in general functional classes**. Significant match to the KOG database (E<10E-5) was used as a guideline for grouping the sequences into the functional classes.

In order to gain insight into the spectrum of molecules present in the *P. perniciosus *midgut, two cDNA libraries from this organ were constructed, sequenced and analysed. The first library was constructed from a pool of midguts from sand flies allowed to feed on sucrose solution (sugar fed). For the construction of the second library (blood fed), midguts from sand flies 4-6 h, 24 h, 2, 3 and 4 days after blood feeding were pooled. These time points cover the course of blood digestion, allowing us to identify molecules transcribed in response to blood feeding. In total, 4511 clones were sequenced and 91% of the sequences were of high quality and included in subsequent analyses. Analysis was performed on 2049 and 2062 sequences for the sugar fed and blood fed libraries, respectively. These sequences were deposited in the NCBI dbEST database under accession numbers [GenBank:GW815603-GW820028]. The comparable number of high quality sequences in each library allows for a better comparison of sequence abundance of specific molecules of interest in the libraries. The bioinformatic analyses of the sequences were performed using the dCAS cDNA annotation software [14]. Sequences were clustered together based on sequence homology and produced 207 and 163 contigs and 712 and 553 singletons (cluster with only one sequence) for the sugar fed and blood fed libraries, respectively. The average sequence per contig ratio was higher in the blood fed library (9.26) than in the sugar fed one (6.46), attributed to the strong induction of certain sequences after blood feeding (such as sequences coding for putative microvillar proteins, proteolytic enzymes and peritrophins, as discussed later). Combining the two libraries produced 370 contigs, 1085 singletons and an average ratio of 8.18 sequences per contig. Most of the clusters (890) had a significant (E<10E-5) BLASTX match to the NCBI non-redundant protein database. However, 565 clusters, mostly singletons, produced no match or low homology and these clusters likely represent transcript coding for novel proteins or potential non-coding regions. Clusters were assigned to general functional classes using the best match BLAST results of the KOG database as a guideline. The overall distribution of clusters in these functional classes in the two libraries is shown in Figure 1. The distribution illustrates the abundance of microvillar proteins and proteins involved in amino acid transport and metabolism (a category including proteolytic enzymes) after blood feeding.

The following paragraphs give a detailed description of the most abundant sequences identified in the libraries and sequences that are of interest with respect to the midgut physiology and *Leishmania *life cycle. The sequences, their putative functions and distribution in the two libraries are listed in Table 1. Table 2 shows the best matches the sequences produced when compared to the NCBI non-redundant protein database using BLASTp.

**Table 1:** Putative function and sequence abundance in the sugar fed (SF) and blood fed (BF) libraries

Cluster	Clone	GenBank	Name	Putative function	SF	BF	Total
46	PPRGUS_P2_F06	EZ933288	PperTryp1	trypsin	513	20	533
16	PPRGUM_P3_G08	EZ933289	PperTryp2	trypsin	10	0	10
63	PPRGFL_P8_E08	EZ933290	PperTryp3	trypsin	0	31	31
81	PPRGFL_P1_E01	EZ933296	PperChym1	chymotrypsin	0	82	82
102	PPRGFL_P7_D06	EZ933297	PperChym2	chymotrypsin	2	11	13
1033	PPRGUM_P1_A02	EZ933298	PperChym3	chymotrypsin	12	0	12
816	PPRGUM_P8_C08	EZ933299	PperChym4	chymotrypsin	7	0	7
710	PPRGUM_P7_F02	EZ933300	PperChym5	chymotrypsin	1	1	2
539	PPRGUL_P4_F08	EZ966131	PperCpepA	carboxypeptidase A	2	1	3
217	PPRGFL_P5_D07	EZ966132	PperCpepB	carboxypeptidase B	1	5	6
126	PPRGUL_P2_A02	EZ966135	PperApeptN	aminopeptidase N	7	13	20
84	PPRGFM_P8_D04	EZ966133	PperAstacin1	astacin	4	8	12
967	PPRGUL_P6_A03	EZ966134	PperAstacin2	astacin	3	0	3
45	PPRGFL_P1_A01	EZ933291	PperMVP1	microvillar protein	0	681	681
40	PPRGFL_P3_G12	EZ933292	PperMVP2	microvillar protein	0	26	26
18	PPRGFL_P5_H01	EZ933293	PperMVP3	microvillar protein	18	6	24
139	PPRGFL_P5_G10	EZ933294	PperMVP4	microvillar protein	0	28	28
52	PPRGFL_P1_E11	EZ933295	PperMVP5	microvillar protein	0	35	35
274	PPRGFL_P7_G05	EZ617708	PperPGRPLB	peptidoglycan recognition protein LB	1	1	2
168	PPRGFL_P5_C02	EZ617707	PperPGRPLC	peptidoglycan recognition protein LC	0	1	1
301	PPRGFL_P8_G01	EZ617706	PperGNBP	gram-negative bacteria binding protein	0	2	2
163	PPRGUM_P7_D04	EZ617709	PperGST1	glutathione-S-transferase Sigma	9	3	12
463	PPRGFM_P5_G03	EZ617710	PperGST2	microsomal glutathione-S-transferase	0	2	2
1322	PPRGUS_P3_F05	EZ617711	PperGST3	glutathione-S-transferase Theta	1	0	1
729	PPRGUM_P1_B05	HM119220	PperPRX	Peroxiredoxin	1	0	1
852	PPRGUS-P1_C06	EZ617712	PperCat	Catalase	9	0	9
892	PPRGUL_P4_B12	EZ617713	PperSOD1	Cu/Zn superoxide dismutase	1	0	1
1166	PPRGUM_P4_E03	EZ617714	PperSOD2	Cu/Zn superoxide dismutase	1	0	1
373	PPRGFM_P2_E03	EZ617715	PperXDH	xanthine dehydrogenase	1	1	2
88	PPRGFM_P2_G07	EZ617716	PperFLC	ferritin light chain	5	6	11
332	PPRGUS-P1_A12	EZ617717	PperFHC	ferritin heavy chain	9	7	16
330	PPRGFS_P8_D12	HM119221	PperPer2	peritrophin	1	4	5
156	PPRGUM_P7_A09	HM119222	PperPer3	peritrophin	1	2	3
97	PPRGFL_P6_E01	EZ933302	PperPer1	peritrophin	0	94	94
124	PPRGFL_P2_E05	EZ933285	PperChit	chitinase	0	3	3
358	PPRGUM_P6_A03	EZ933286	cluster 358	PM formation/unknown	10	3	13
379	PPRGFM_P6_A06	EZ933287	cluster 379	PM formation/unknown	2	4	6
174	PPRGFL_P4_A04	HQ015444	PperGH13	glycoside hydrolyse	23	9	32
183	PPRGFM_P6_G03	HQ015443	PperGH31	glycoside hydrolyse	12	3	15
79	PPRGFL_P6_G10	HQ015441	cluster 79	lipid recognition/unknown	0	9	9
652	PPRGUS_P7_D02	HQ015442	PperSA	40S ribosomal protein SA	8	1	9
461	PPRGUM_P6_G08	EZ933301	PperS7	ribosomal protein S7	6	2	8

**Table 2:** Selected clusters of combined *P. perniciosus *midgut cDNA libraries: best match to the NCBI non-redundant protein database

Cluster	GenBank	Name	Best match to nr protein database	Best match GenBank	NR E value
46	EZ933288	PperTryp1	trypsin 2 [Lutzomyia longipalpis]	ABM26905.1	3.00E-82
16	EZ933289	PperTryp2	trypsin 1 [Phlebotomus papatasi]	AAM96940.1	3.00E-76
63	EZ933290	PperTryp3	putative trypsin 3 [Lutzomyia longipalpis]	ABV60308.1	1.00E-92
81	EZ933296	PperChym1	putative chymotrypsin [Lutzomyia longipalpis]	ABV60294.1	1.00E-109
102	EZ933297	PperChym2	chymotrypsin-like protein [Phlebotomus papatasi]	ABV44728.1	4.00E-94
1033	EZ933298	PperChym3	putative chymotrypsin [Lutzomyia longipalpis]	ABV60294.1	8.00E-77
816	EZ933299	PperChym4	putative chymotrypsin [Lutzomyia longipalpis]	ABV60293.1	6.00E-58
710	EZ933300	PperChym5	serine protease1/2 [Culex quinquefasciatus]	XP_001845462.1	1.00E-59
539	EZ966131	PperCpepA	carboxypeptidase A [Aedes aegypti]	AAT36730.1	1.00E-116
217	EZ966132	PperCpepB	carboxypeptidase B-like protein [Phlebotomus papatasi]	ABV44754.1	1.00E-170
126	EZ966135	PperApeptN	aminopeptidase N [Aedes aegypti]	AAK73351.1	1.00E-44
84	EZ966133	PperAstacin1	astacin-like metalloprotease [Lutzomyia longipalpis]	ABV60299.1	1.00E-92
967	EZ966134	PperAstacin2	AGAP010758-PA [Anopheles gambiae]	XP_318553.4	2.00E-49
45	EZ933291	PperMVP1	microvillar-like protein 1 [Lutzomyia longipalpis]	ABV60289.1	2.00E-69
40	EZ933292	PperMVP2	microvilli-like protein 2 [Phlebotomus papatasi]	ABV44759.1	7.00E-69
18	EZ933293	PperMVP3	microvilli-like protein 3 [Phlebotomus papatasi]	ABV44760.1	6.00E-57
139	EZ933294	PperMVP4	microvilli-like protein [Phlebotomus papatasi]	ABV44761.1	2.00E-92
52	EZ933295	PperMVP5	microvillar-like protein [Lutzomyia longipalpis]	ABV60295.1	1.00E-67
274	EZ617708	PperPGRPLB	putative PGRP [Phlebotomus papatasi]	ABV60369.1	1.00E-104
168	EZ617707	PperPGRPLC	PGRP-lc isoform [Anopheles gambiae]	AGAP005203-PC	4.00E-62
301	EZ617706	PperGNBP	GNBP [Aedes aegypti]	XP_001664288	1.00E-77
163	EZ617709	PperGST1	GST-like protein [Phlebotomus papatasi]	ABV44736.1	3.00E-113
463	EZ617710	PperGST2	microsomal GST [Culex quinquefasciatus]	XP_001863047.1	8.00E-20
1322	EZ617711	PperGST3	GST theta [Aedes aegypti]	XP_001659667.1	8.00E-22
729	HM119220	PperPRX	peroxiredoxin-like [Phlebotomus papatasi]	ABV44727.1	6.00E-86
852	EZ617712	PperCat	putative catalase [Lutzomyia longipalpis]	ABV60342.1	0.00E+00
892	EZ617713	PperSOD1	putative Cu/Zn SOD [Lutzomyia longipalpis]	ABV60343.1	5.00E-89
1166	EZ617714	PperSOD2	superoxide dismutase [Culex quinquefasciatus]	XP_001866335	9.00E-64
373	EZ617715	PperXDH	XDH [Lutzomyia longipalpis]	CAP08999.1	4.00E-45
88	EZ617716	PperFLC	FLC-like [Phlebotomus papatasi]	ABV44741.1	1.00E-111
332	EZ617717	PperFHC	FHC-like [Phlebotomus papatasi]	ABV44737	7.00E-73
330	HM119221	PperPer2	putative peritrophin [Lutzomyia longipalpis]	ABV60320.1	2.00E-09
156	HM119222	PperPer3	peritrophin-like protein [Phlebotomus papatasi]	ABV44751.1	4.00E-59
97	EZ933302	PperPer1	peritrophin-like protein [Phlebotomus papatasi]	ABV44705.1	1.00E-102
124	EZ933285	PperChit	midgut chitinase [Phlebotomus papatasi]	AAV49322.1	4.00E-61
358	EZ933286	cluster 358	14.5 kDa salivary protein [Phlebotomus duboscqi]	ABI20163	3.00E-49
379	EZ933287	cluster 379	31.5 kDa midgut protein [Phlebotomus papatasi]	ABV44721.1	5.00E-90
174	HQ015444	PperGH13	alpha-amylase [Aedes aegypti]	XP_001649787.1	1.00E-170
183	HQ015443	PperGH31	GK14321 [Drosophila willistoni]	XP_002073831.1	0.00E+00
79	HQ015441	cluster 79	Niemann-Pick Type C2, putative [Aedes aegypti]	XP_001647805.1	2.00E-14
652	HQ015442	PperSA	40S ribosomal protein SA [Simulium nigrimanum]	ACZ28384.1	1.00E-68
461	EZ933301	PperS7	40S ribosomal protein S7-like protein [Phlebotomus papatasi]	ABV44745.1	2.00E-95

### Trypsins

**Figure 2:**
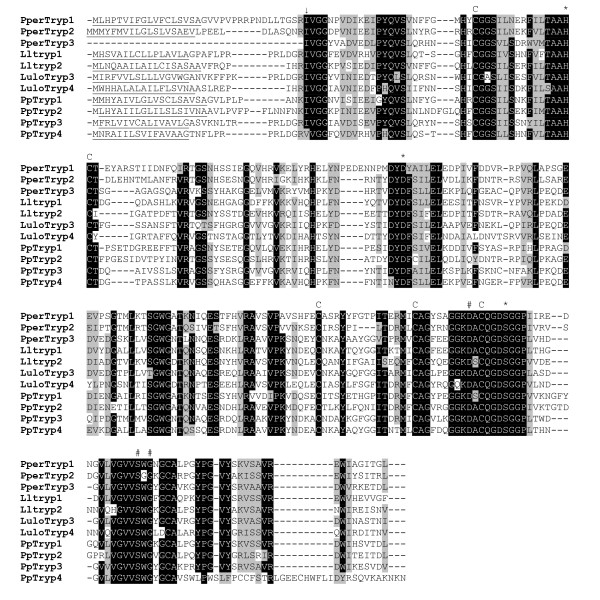
**Multiple sequence alignment of putative sand fly trypsins**. Pper: *Phlebotomus pernicios*us, Pp: *Phlebotomus papatasi*, Lulo: *Lutzomyia longipalpis*. Predicted signal peptides are underlined, the putative activation cleavage site is indicated by (↓), conserved cysteines (C), catalytic H/D/S residues marked by (*) and substrate binding site marked by (#). Accession numbers: PperTryp1 [GenBank:EZ933288], PperTryp2 [GenBank:EZ933289], PperTryp3 [GenBank:EZ933290], Lltryp1 [GenBank:ABM26904], Lltryp2 [GenBank:ABM26905], LuloTryp3 [GenBank:ABV60308], LuloTryp4 [GenBank: ABV60300], PpTryp1 [GenBank:AAM96940], PpTryp2 [GenBank:AAM96941], PpTryp3 [GenBank:AAM96942], PpTryp4 [GenBank:AAM96943].

Proteolytic enzymes were among the most abundant sequences detected in the libraries. Three putative trypsins were identified. *PperTryp1 *[GenBank:EZ933288], cluster 46, was one of the most abundant transcripts overall, strongly overrepresented in the sugar fed library (513 of 533 sequences). The putative protein has a predicted molecular weight of 27.6 kDa after cleavage of the signal peptide and a pI of 5.41. *PperTryp2 *[GenBank:EZ933289], cluster 16, is a less abundant (10 sequences) putative trypsin that was only detected in the sugar fed library. The putative mature protein has a predicted molecular weight of 26.9 kDa and a high pI of 8.83 (similar to a putative *P. papatasi *trypsin, PpTryp3 [GenBank:AAM96942]. Sequences coding for a third putative trypsin named *PperTryp3 *[GenBank:EZ933290] (cluster 63, 5' truncated) originated from the blood fed midgut library. In blood fed midguts we also identified a few partial transcripts, coding for a putative variant of this protein (5 sequences represented by clone PPRGFL_P8_H08, [GenBank:GW817404], Cluster 61). This Cluster 61 variant shows 82% identity to PperTryp3 at the amino acid level. Multiple sequence alignment of the putative *P. perniciosus *trypsin molecules (Figure 2) shows that structural cysteines, the H/D/S catalytic triad and putative substrate specifying residues are well conserved. Both *PperTryp1 *and *PperTryp2*, for which we obtained the full-length sequence of the transcripts, are pre-pro-peptides; having a predicted signal peptide and a putative pro-peptide cleavage site for activation of the mature protein.

**Figure 3:**
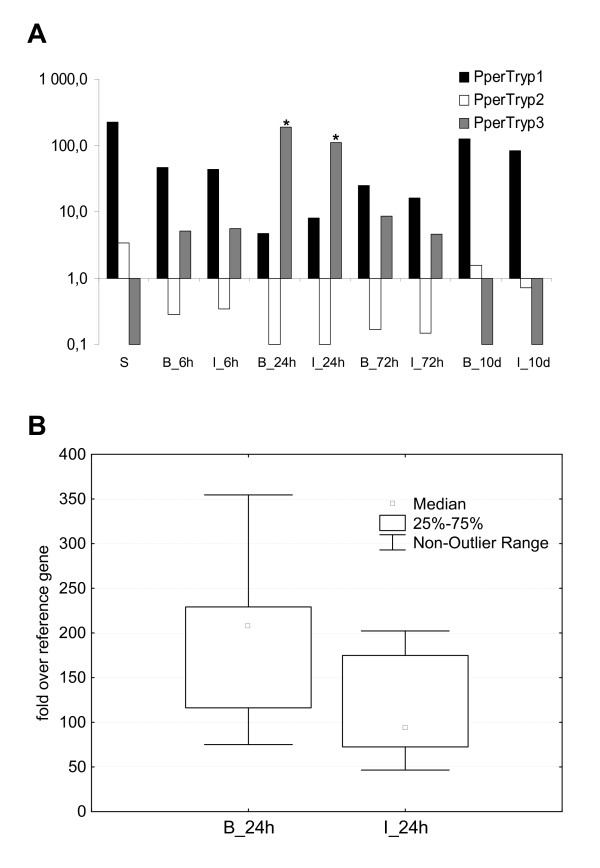
**Influence of blood feeding and *L. infantum *infection on the dynamics of *P. perniciosus *trypsins expression**. (A) The graph shows PperTryp1, PperTryp2 and PperTryp3 expression as fold over the reference housekeeping gene (PpPerS7 ribosomal protein) before and after the blood feeding (6 hours, 24 hours, 72 hours and 10 days). Each column represents the mean of ten females. S, sugar fed sand flies; B, blood fed sand flies; I, blood fed and *L. infantum *infected sand flies. The statistically significant difference between the infected and uninfected sand flies is indicated by (*). (B) The graph shows significant difference of PperTryp3 expression in uninfected (B_24) and infected (I_24) sand flies 24 hours after blood feeding; Mann-Whitney U Test (U = 20; Z = 2.268), p = 0.023.

In order to describe the expression dynamics of the identified putative trypsin molecules, we performed a qPCR analysis of the three transcripts before, and at several time points after, blood feeding. The results (Figure 3) correlate with the sequence abundance in the two libraries, proving the validity of the library comparison approach. In addition, the qPCR analysis provides a more detailed view of the trypsin expression after blood feeding. *PperTryp1*, the most abundant trypsin identified, was down regulated as soon as 6 h after blood feeding and further suppressed 24 h post-blood meal (about 1/50^th ^of pre-blood meal levels). Its expression returned to the pre-blood meal levels in the sand flies that had passed the remnants of blood meal. *PperTryp2 *was detected in lower amounts (about 1/70^th ^of PperTryp1) and represents another trypsin down regulated by blood feeding, with a time course similar to that observed for *PperTryp1*. In contrast, the qPCR analysis confirmed *PperTryp3 *as the main blood feeding-induced trypsin molecule. *PperTryp3 *expression was already elevated after 6 h and the highest quantity of the transcripts was observed 24 h post-blood meal. *PperTryp3 *returned to negligible amounts in sand flies that had finished blood digestion.

**Figure 4:**
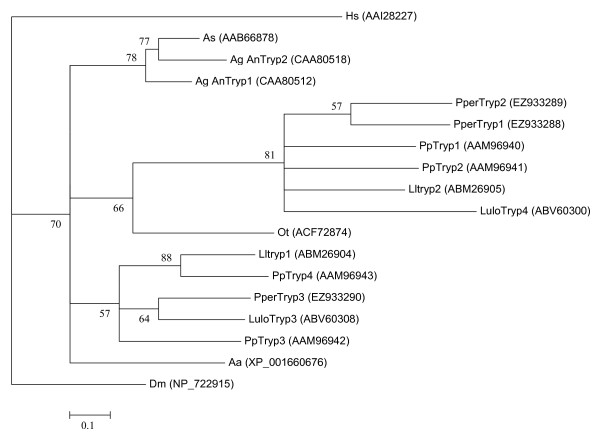
**Phylogenetic analysis of putative trypsins from *Anopheles stephensi *(As), *Ochlerotatus taeniorhynchus *(Ot), *Homo sapiens *(Hs), *Aedes aegypti *(Aa), *Anopheles gambiae *(Ag), *Phlebotomus perniciosus *(Pper), *Lutzomyia longipalpis *(Lulo), *Phlebotomus papatasi *(Pp) and *Drosophila melanogaster *(Dm)**. GenBank accession numbers are given in parentheses and node support is indicated by the bootstrap values.

This study brings the first expression analysis of sand fly trypsins using precise quantification by the means of qPCR. The observed *P. perniciosus *trypsin expression profile is in accordance with the results of earlier studies of *P. papatasi *and *L. longipalpis *midgut trypsin abundance from data acquired by semi-quantitative end-point PCR [15,16] and comparison of transcript abundance in cDNA libraries before and after blood feeding [5,6]. In all the three species, one or several trypsin transcripts (see Figure 4 for sequence accession numbers) are present in high abundance in sugar fed females while their quantities decrease after the intake of blood (*PperTryp1*, *PperTryp2*, *LlTryp2*, *PpTryp1*, *PpTryp2*). At the same time, the expression of other putative trypsins (*PperTryp3*, *LlTryp1 *and *PpTryp4*) is induced upon blood feeding.

Phylogenetic analysis of the putative trypsins (Figure 4) shows that the sequences abundant before blood feeding share similarity and, together with LuloTryp4 (reported in similar numbers both from blood fed and sugar fed *L. longipalpis *sand flies), form a clade apart from the other sand fly trypsins that include PperTryp3. The predicted pre-pro-peptide structure and high abundance of transcripts in sugar fed sand flies together with the virtual absence of trypsin-like enzymatic activity in sugar fed sand fly midguts [17] suggests that PperTryp1, PperTryp2 and their respective homologs are associated with initial blood meal digestion as they can be quickly translated and processed following blood feeding.

The onslaught of proteolytic activity after the intake of blood is one of the barriers for *Leishmania *development in the midgut [2]. Sant'Anna et al. [10] have shown that suppression of the major blood meal-induced trypsin (LlTryp1) in *L. longipalpis *by the means of RNAi enhances the survival of *L. mexicana *in the midgut. Some studies have demonstrated the ability of *L. major *and *L. infantum *to suppress or delay the peak of trypsin activity in the midgut [18-20]. Transcriptomic studies [5,6] have also shown modulation of trypsin-like transcript abundance in *P. papatasi *and *L. longipalpis *midgut in the presence of *Leishmania *parasites. qPCR was used to measure trypsin expression in infected sand flies to evaluate modulation of the *P. perniciosus *midgut trypsins by *L. infantum*. Our analysis showed that the amount of *PperTryp3*, the major blood meal-induced trypsin, is decreased in the presence of *L. infantum *(Figure 3). This difference was observed in sand flies 24 hours post-blood meal, which correlated with the peak of *PperTryp3 *expression in uninfected blood fed sand flies. Our findings suggest the ability of *L. infantum *to suppress or delay the expression of the major blood-induced trypsin in *P. perniciosus *and identify this molecule as an interesting candidate for future studies.

### Chymotrypsins

**Figure 5:**
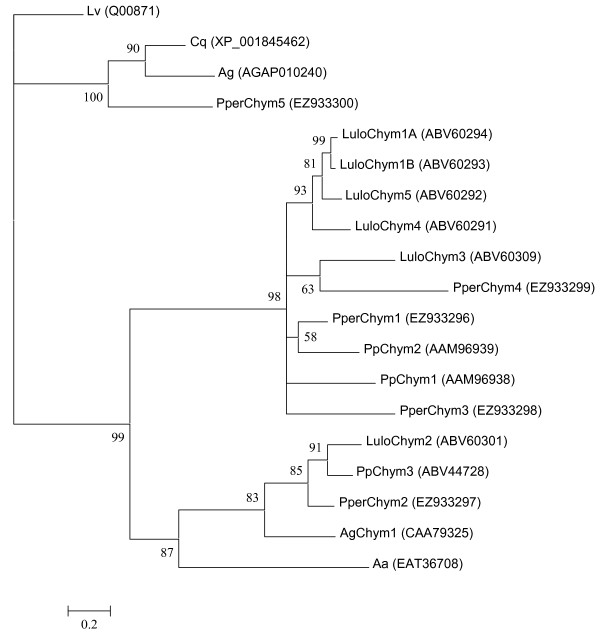
**Phylogenetic analysis of putative chymotrypsin molecules from *Litopenaeus vannamei *(Lv), *Culex quinquefasciatus *(Cq), *Anopheles gambiae *(Ag), *Aedes aegypti *(Ae), *Phlebotomus perniciosus *(Pper), *Lutzomyia longipalpis *(Lulo) and *Phlebotomus papatasi *(Pp)**. GenBank accession numbers are given in parentheses and node support indicated by the bootstrap value.

**Figure 6:**
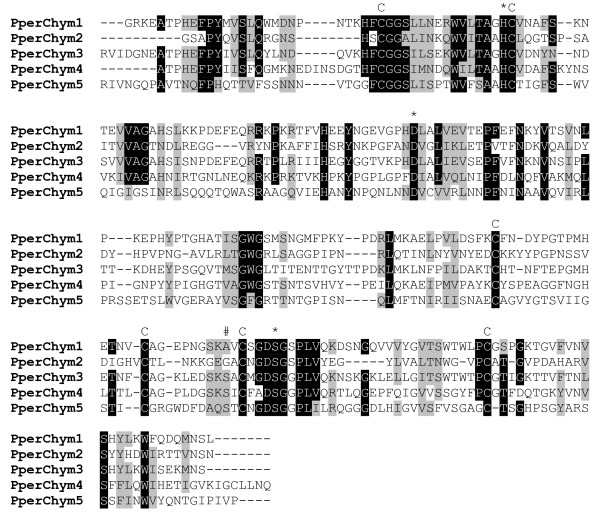
**Multiple sequence alignment of putative *P. perniciosus *chymotrypsins (partial sequences)**. Conserved cysteines are indicated (C), catalytic H/D/S residues marked by (*) and a serine residue implicated in chymotrypsin substrate specificity marked by (#). Accession numbers: PperChym1 [GenBank:EZ933296], PperChym2 [GenBank:EZ933297], PperChym3 [GenBank:EZ933298], PperChym4 [GenBank:EZ933299], PperChym5 [GenBank:EZ933300].

Chymotrypsin-like enzymes are another group of proteases found in abundance in the midgut of sand flies and mosquitoes. Five clusters coding for putative chymotrypsins were identified in the libraries and each cluster was 5' truncated. *PperChym1 *[GenBank:EZ933296], cluster 81, was the most abundant and was only detected in the blood fed midgut library (82 sequences). Similarly, the second most abundant chymotrypsin-like sequence, *PperChym2 *[GenBank:EZ933297], cluster 102, probably codes for a digestive enzyme up-regulated by blood feeding as 11 of the 13 sequences were found in the blood fed library. In contrast, *PperChym3 *[GenBank:EZ933298], cluster 1033, and *PperChym4 *[GenBank:EZ933299], cluster 816, sequences were only found in the sugar fed sand fly library. The expression pattern of these chymotrypsins is similar to the aforementioned trypsin molecules indicating that there may be early and late classes of serine proteases in sand flies, similar to what has been observed in mosquitoes[21] The least abundant putative chymotrypsin sequence *PperChym5 *[GenBank:EZ933300], cluster 710, was represented by one sequence in each of the libraries. The phylogenetic analysis of amino acid sequences (Figure 5) shows conservation in sequence homology of PperChym1-4 with putative *P. papatasi *and *L. longipalpis *midgut chymotrypsins. PperChym2 formed a subclade along with LuloChym2 and Ppchym3. PperChym5 is more distantly related to putative chymotrypsins described in other sand flies. The most similar sequence in the NCBI non-redundant protein database is a putative serine protease of *Culex quinquefasciatus *[GenBank:XP_001845462.1] (E = 1 e-59) and the best match in the Swissprot database is the white shrimp, *Litopenaeus vannamei*, Chymotrypsin BI [Swiss-Prot: Q00871, E = 3 e-36] with a proven chymotrypsin catalytic activity. Also, a conserved serine residue at the substrate specifying site suggests a chymotrypsin-like specificity of PperChym5 enzyme (Figure 6). The H/D/S catalytic triad and cysteine residues are well conserved among all the putative *P. perniciosus *chymotrypsin sequences. Putative chymotrypsin transcript abundance has previously been shown to be altered by *Leishmania *infection in the midgut. *LuloChym1A *in *L. longipalpis *and *PpChym2 *in *P. papatasi *were reported as underrepresented in the midgut in the presence of *L. infantum *and *L. major*, respectively[5,6].

### Carboxypeptidases

**Figure 7:**
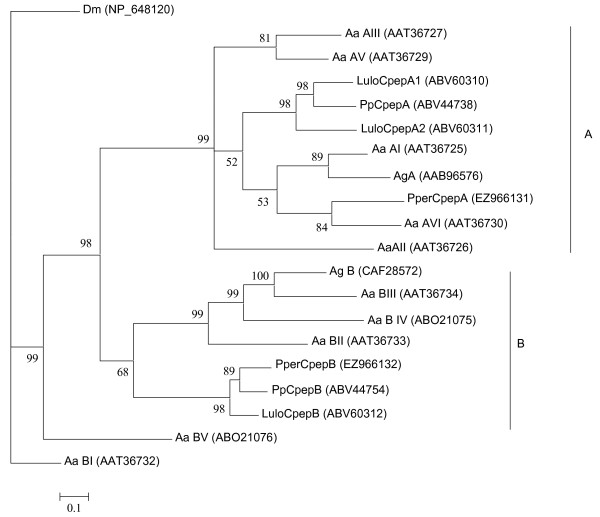
**Phylogenetic analysis of putative carboxypeptidases from *Drosophila melanogaster *(Dm), *Aedes aegypti *(Aa), *Anopheles gambiae *(Ag), *Phlebotomus perniciosus *(Pper), *Lutzomyia longipalpis *(Lulo) and *Phlebotomus papatasi *(Pp)**. Genbank accession numbers are given in parentheses and node support is indicated by the bootstrap values.

**Figure 8:**
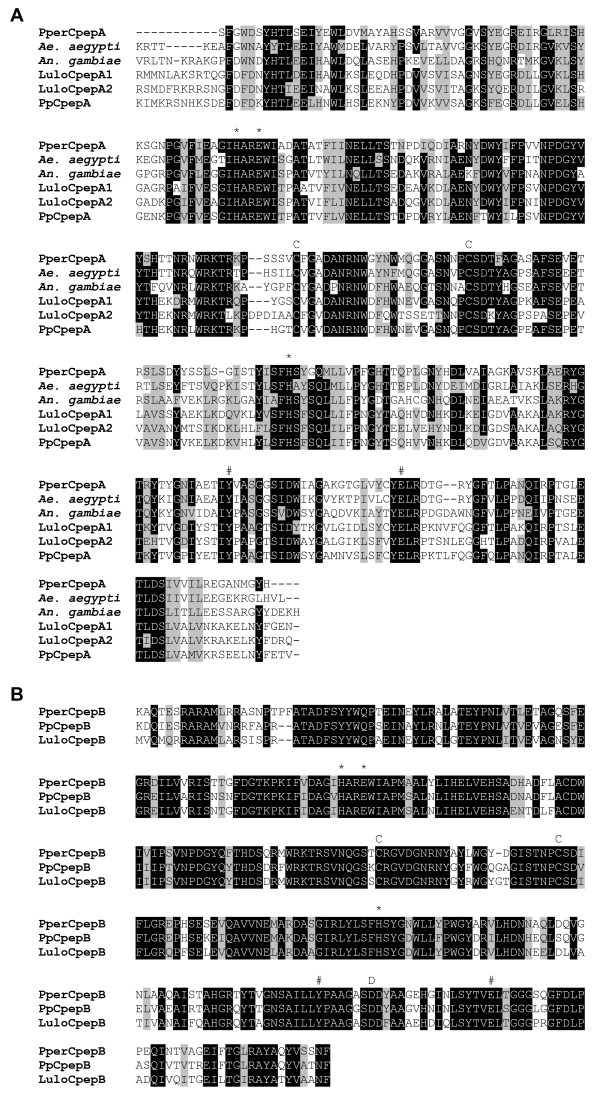
**Sequence alignment of putative midgut carboxypeptidases**. (A) Comparison of mature Carboxypeptidase A proteins of *Aedes aegypti *(*A. aegypti*), *Anopheles gambiae *(*A. gambiae*), *Phlebotomus perniciosus *(Pper), *Phlebotomus papatasi *(Pp) and *Lutzomyia longipalpis *(Lulo). N-terminal portion of the peptides are not shown due to PperCpepA 5' mRNA truncation. Conserved cysteines are indicated (C), metal binding residues are marked by (*) and catalytic residues are marked by (#). (B) Comparison of mature Carboxypeptidase B proteins of *P. perniciosus *(Pper), *P. papatasi *(Pp) and *L. longipalpis *(Lulo). Conserved cysteines are indicated (C), metal binding residues are marked by (*), catalytic residues are marked by (#) and a conserved aspartate in the binding pocket of carboxypeptidases B is indicated (D). Accession numbers: PperCpepA [GenBank:EZ966131], *A. aegypti *[GenBank:AAT36730], *A. gambiae *[GenBank:AAB96576], LuloCpepA1 [GenBank:ABV60310], LuloCpepA2 [GenBank:ABV60311], PpCpepA [GenBank:ABV44738], PperCpepB [GenBank:EZ966132], PpCpepB [GenBank:ABV44754], LuloCpepB [GenBank:ABV60312].

A number of clusters coding for putative proteins with homology to carboxypeptidases were identified. Two putative metallo-carboxypeptidases of the M14 A/B subfamily were named *PperCpepA *and *PperCpepB. PperCpepA *[GenBank:EZ966131] (cluster 539, 5' truncated) shows similarity to carboxypeptidases A described from mosquitoes. Phylogenetically, PperCpepA clusters more distantly from the carboxypeptidases A described in the midgut of other phlebotomine species (Figure 7). Carboxypeptidase A, a zinc-metalloprotease, can hydrolyze aromatic and aliphatic side chains from the C-terminus. *PperCpepB *[GenBank:EZ966132] (cluster 217, 5' truncated) is similar to mosquito and sand fly midgut carboxypeptidase B. Carboxypeptidase B specifically hydrolyzes C-terminal arginine and lysine. PperCpepB possesses the conserved aspartate residue at the position responsible for this specific substrate recognition [22] (Figure 8). Due to the low number of sequences in this cluster a comparative analysis between the sugar fed and blood fed libraries was not possible; however, it is notable that five of the six sequences of *PperCpepB *were contributed by the blood fed library. *Anopheles gambiae *midgut carboxypeptidase B has been shown to be up-regulated by *Plasmodium *infection and antibodies against one of these enzymes, CPBAg1 [GenBank:CAF28572] blocked parasite development in the mosquito midgut [23]. In *L. longipalpis*, one of the carboxypeptidases transcripts, LuloCpepA1, [GenBank:ABV60310] was underrepresented in a cDNA library from *L. infantum*-infected midgut as compared to uninfected sand flies [6].

### Aminopeptidases

A partial transcript coding for a putative alanyl aminopeptidase was identified (cluster 126). The molecule, named *PperApeptN *[GenBank:EZ966135], is similar to mosquito membrane aminopeptidases of the M1 family (aminopeptidase N). It was abundant in both libraries (13 and 7 sequences in blood fed and sugar fed midgut libraries, respectively). Membrane alanyl aminopeptidases were described in the midgut of many Dipteran species including mosquitoes, where they were identified as receptors for *Plasmodium *ookinetes and also *Bacillus thuringiensis *Cry toxin binding [24,25]. In the sand fly midgut, aminopeptidase activity was detected after blood feeding, mainly associated with the midgut wall (using leucine-p-nitroanilide LpNA as a substrate) [17]. This activity was reduced in *P. papatasi *and *P. langeroni *following infection with *L. major *[19].

### Astacins

**Figure 9:**
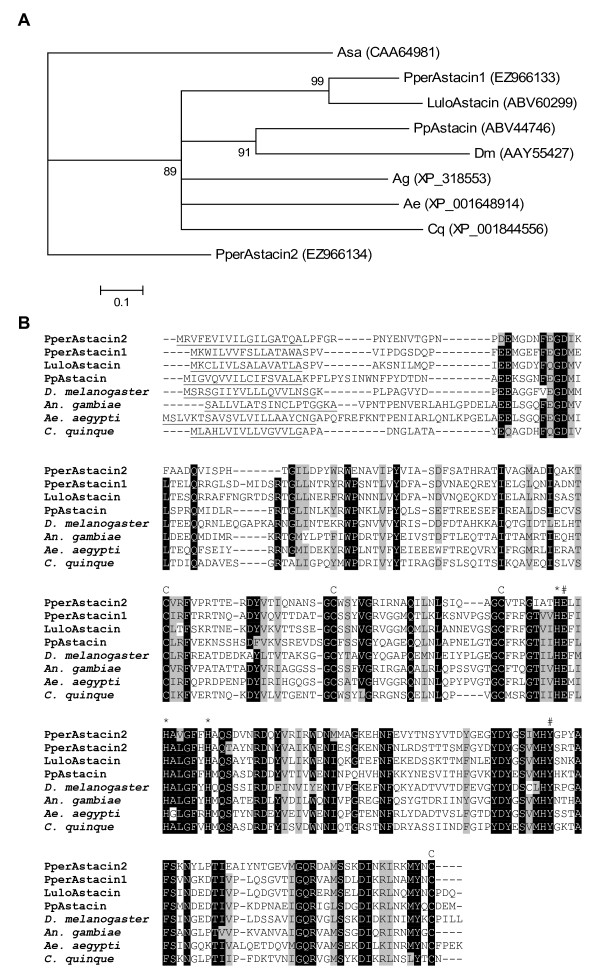
**Phylogenetic analysis and sequence alignment of (putative) astacins**. (A) *Astacus astacus *(Asa), *Drosophila melanogaster *(Dm), *Aedes aegypti *(Ae), *Anopheles gambiae *(Ag), *Phlebotomus perniciosus *(Pper), *Lutzomyia longipalpis *(Lulo), *Phlebotomus papatasi *(Pp) and *Culex quinquefasciatus *(Cq). Accession numbers are given in parentheses and node support is indicated by the bootstrap values. (B) *Drosophila melanogaster *(D. melanogaster), *Aedes aegypti *(*A. aegypti*), *Anopheles gambiae *(*A. gambiae*), *Phlebotomus perniciosus *(Pper), *Lutzomyia longipalpis *(Lulo), *Phlebotomus papatasi *(Pp) and *Culex quinquefasciatus *(*C. quinque*). Predicted signal peptide is underlined, conserved cysteines are indicated (C), the metal binding residues marked by (*) and catalytic residues marked by (#). Accession numbers: PperAstacin2 [GenBank:ABV44746], PperAstacin1 [GenBank:EZ966133], LuloAstacin [GenBank:ABV60299], PpAstacin [GenBank:ABV44746], *D. melanogaster *[GenBank:AAY55427], *A. gambiae *[GenBank:XP_318553], *A. aegypti *[GenBank:XP_001648914], *C. quinque *[GenBank:XP 001844556].

Two clusters coding for putative astacin-like zinc metalloproteases were identified in the libraries. The more abundant cluster, *PperAstacin1 *[GenBank:EZ966133], cluster 84, is predicted to encode a protein with a molecular weight of 27.0 kDa once secreted and pI of 5.05. It was present both in the sugar fed and blood fed libraries. The transcript of cluster 967 was named *PperAstacin2 *[GenBank:EZ966134] and the predicted translated product has a molecular weight of 26.5 and pI 6.00 after cleavage of the signal peptide. It was only detected in the sugar fed library. Phylogenetic analysis of other putative astacin sequences shows that PperAstacin1 is similar to astacin-like molecules previously described in *L. longipalpis*, LuloAstacin, [GenBank:ABV60299] *P. papatasi *and other Diptera. PperAstacin2 is most similar to a putative astacin from *A. gambiae *using BLASTp similarity search of the NCBI non-redundant protein database. However, in a phylogenetic analysis it branches away from all other Dipteran sequences (Figure 9A). Multiple sequence alignment (Figure 9B) shows the differences in amino acid sequences and illustrates the conservation of all residues likely responsible for zinc-binding and catalytic activity in the putative *P. perniciosus *astacins.

### Microvillar proteins

The most abundant transcripts identified in the library were sequences coding for proteins with similarity to major insect allergen proteins. These insect-specific proteins containing insect-allergen domains [InterPro:IPR010629] were first described as the major human allergens in the faeces of the cockroaches *Blatella germanica *and *Periplaneta americana *[26]. In butterflies of the Pieridae family, a novel family of proteins with multiple insect-allergen domains has evolved (nitrile-specifier protein family) to serve a role in detoxification of plant metabolites in the butterfly larvae food [27,28]. In mosquitoes, proteins with a single insect-allergen domain have been identified and termed G12 microvillar proteins. These molecules have been shown to be induced in the mosquito midgut after blood-feeding [29,30]. In *Aedes aegypti*, the G12 protein, AEG12, [GenBank:AAL05408.1] has been shown to be expressed only in the midgut after blood feeding and located on the microvillar membranes of the midgut epithelial cells [30]. The role of insect allergen proteins, other than nitrile-specifier protein family in Pieridae, has not yet been characterized.

**Figure 10:**
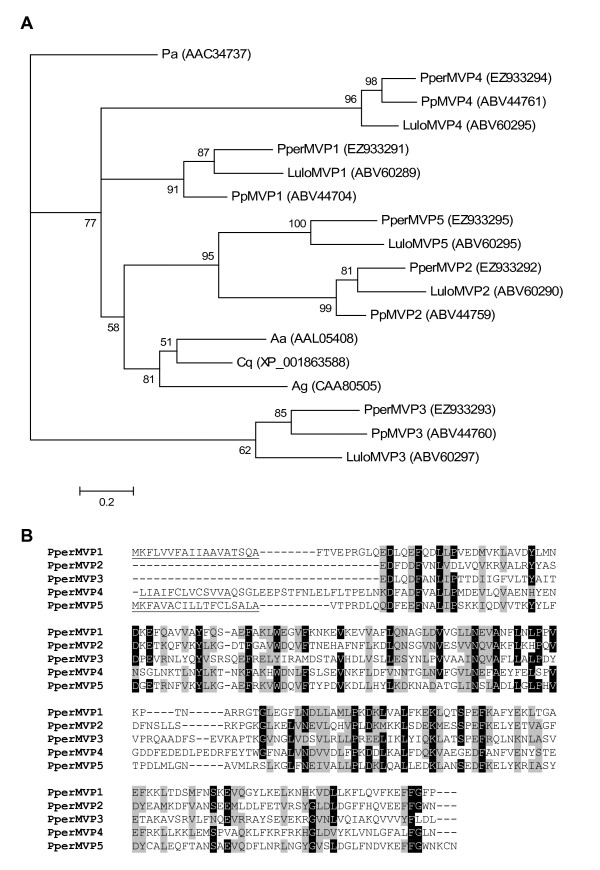
**Phylogenetic analysis and sequence alignment of putative microvillar proteins**. (A) *Periplaneta americana *(Pa), *Phlebotomus perniciosus *(Pper), *Phlebotomus papatasi *(Pp), *Lutzomyia longipalpis *(Lulo), *Aedes aegypti *(Ae), *Culex quinquefasciatus *(Cq) and *Anopheles gambiae *(Ag). Accession numbers are given in parentheses. Node support is indicated by the bootstrap values. (B) *Phlebotomus perniciosus *(Pper). The predicted signal peptides are underlined. Accession numbers: PperMVP1 [GenBank:EZ933291], PperMVP2 [GenBank:EZ933292], PperMVP3 [GenBank:EZ933293], PperMVP4 [GenBank:EZ933294], PperMVP5 [GenBank:EZ933295].

We identified five putative homologs of the insect-allergen proteins in the *P. perniciosus *libraries. These putative microvillar proteins (MVPs) possess a predicted signal peptide (where full-length sequences were obtained) and a single insect-allergen domain. *PperMVP1 *[GenBank:EZ933291] encodes a putative protein with a mature molecular weight of 21.4 kDa and pI of 5.16. Derived from 681 sequences (cluster 45 and variants) *PperMVP1 *was the most abundant transcript overall. Transcripts of *PperMVP1*, and three other *P. perniciosus *MVPs, were only found in the blood fed cDNA library. These other blood feeding induced MVPs include *PperMVP2 *[GenBank:EZ933292] (cluster 40 and variants, sequence 5' truncated), *PperMVP4 *[GenBank:EZ933294] (cluster 139, 5' truncated) and *PperMVP5 *(EZ933295, cluster 52 and variants; 22 kDa, pI 4.77). PperMVP4 amino acid sequence, although truncated, contains predicted glycosylation sites (3 N-glycosylations). The only MVP transcript overrepresented in the sugar fed library is *PperMVP3 *[GenBank:EZ933293] (cluster 52 and variants, 5' truncated). As demonstrated in the phylogenetic analysis (Figure 10A), the identified sequences show high similarity to the respective five MVPs previously identified in the midgut of *L. longipalpis*. Homologs of four of these proteins are also known in *P. papatasi *(PpMVP1-4; Figure 10A). Interestingly, no sequence with high similarity to PperMVP5 was found in the midgut of *P. papatasi*. The phylogenetic tree also shows that PperMVP3 and its putative orthologues LuloMVP3 and PpMVP3 clade away from all the other sand fly and mosquito MVPs. This is in accordance with the fact that all the three seem to be down-regulated be blood feeding unlike other MVPs. Multiple sequence alignment (Figure 10B) shows that the five putative *P. perniciosus *MVPs share little sequence homology suggesting that these molecules may have different functions altogether.

### Antimicrobial molecules

Several transcripts encode proteins putatively involved in the immune response of the sand fly midgut. Two clusters coding for putative peptidoglycan recognition proteins (PGRPs) were identified. PGRPs play central and diverse roles in activating insect immune reactions including the melanization cascade, phagocytosis, and signal transduction pathways for production of antibacterial peptides. *PperPGRPLB *[GenBank:EZ617708], cluster 274, is similar to a PGRP previously reported from *L. longipalpis *(*LuloPGRP*) [GenBank:ABV60332], and *P. papatasi *(*PpPGRP*) [GenBank:60369] midgut. Although the transcript appears to be 5' truncated, based on homology to other full length sand fly transcripts we predict it to be a protein of about 22 kDa. A homologous (but secreted) protein PGRPLB in *Drosophila melanogaster *[GenBank:AAN13505], mainly expressed in the midgut, was shown to regulate the Imd signalling pathway controlling the immune response against Gram-negative bacteria [31]. An *A. gambiae *PGRPLB homolog [GenBank:EAA01800] is induced by both bacterial and *Plasmodium *infections [32]. *PperPGRPLC *[GenBank:EZ617707], cluster 168, transcript 5' truncated encodes a putative protein with similarity to insect PGRPLC proteins that act as membrane-localized peptidoglycan receptors activating the Imd pathway. PGRPLC-like molecules have not been previously reported in sand flies. In searching the midgut transcriptomes of sand flies, partial transcripts with homology to *PperPGRPLC *were found in *P. papatasi *[GenBank:ES347179] and *L. longipalpis *[GenBank:AM098991]. In *A. gambiae*, PGRPLC [GenBank:AGAP005203] signalling controls the size of symbiotic bacteria populations, intestinal bacterial infections and *Plasmodium *infections [33].

In addition to PGRPs, a transcript encoding a putative gram-negative bacteria binding protein was identified and named *PperGNBP *[GenBank:EZ617706], cluster 301, transcript 5' truncated. Gram-negative bacteria-binding proteins serve as pattern recognition receptors binding to pathogen-associated beta-1,3-glucans in insects and they have been shown to play a role in mosquito defence against bacteria and *Plasmodium *infection [34].

It is likely that the identified sand fly pattern recognition proteins are involved in protection against bacteria in the midgut; however, similar to the mosquito homologs, they might also have an impact on *Leishmania *infection. Interestingly, Kumar et al. [35] have recently described a novel secreted peroxidase/dual oxidase system in *A. gambiae *midgut that catalyses cross-linking of a dityrosine network on the luminal surface of the epithelial cells. This network decreases the permeability of the mucus layer to immune elicitors and thus prevents induction of PGRPs, nitric oxide synthase and other immune responsive genes. Silencing of the peroxidase/dual oxidase system causes a drastic reduction in *Plasmodium *infection in the midgut [35]. We have not identified any homologs of the components of the peroxidase/dual oxidase system in *P. perniciosus *midgut in this analysis. Further studies are needed to see whether a dityrosine barrier is formed in the sand fly midgut. In our analysis, we did not detect any antimicrobial peptide transcripts in the midgut of *P. perniciosus*, although a defensin was previously characterized in *Phlebotomus duboscqi *midgut [36] and defensin transcripts were also reported from the midgut of *L. longipalpis *[6,7].

### Oxidative stress molecules

A number of transcripts were identified coding for putative antioxidant enzymes. In hematophagous insects, blood meal-derived free heme is a strong pro-oxidant and can tax the midgut antioxidant system. In addition to their protective role, redox-related molecules were shown to regulate midgut epithelial immunity and impact the outcome of bacterial and parasitic infections in mosquitoes [35,37]

### Glutathione S-tranferases (GSTs) and peroxiredoxin

Putative components of the glutathione-thioredoxin system, the central redox homeostasis maintaining pathway in insects, were found [38,39]. Several transcripts coding for putative glutathione-S-transferases (GSTs), enzymes catalyzing substrate detoxification by a thiol tripeptide glutathione, were identified in both libraries. *PperGST1 *[GenBank:EZ617709], cluster 163, encodes a putative intracellular GST of the Sigma subfamily. The putative protein is predicted to be 23.2 kDa and have a pI of 5.00. It is nearly identical to putative Sigma GSTs described from the midgut of *P. papatasi *[GenBank:ABV44736] (98% identity) and *L. longipalpis *LuloGST1 [GenBank:ABV60329] (97% identity). *PperGST2 *[GenBank:EZ617710] (cluster 463, 5' truncated) encodes a putative transmembrane protein that has homology to microsomal GSTs of the MAPEG super family. Homologs of *PperGST2 *were found in the EST databases of *P. papatasi *[GenBank:FK811479] and *L. longipalpis *[GenBank:EW990920]. *PperGST3 *[GenBank:EZ617711] (cluster 1322, 5' truncated) shares similarity with other Dipteran GSTs of the Theta class, a class not previously reported in sand flies. A homolog of PperGST3 was identified by searching the *L. longipalpis *whole fly cDNA library [GenBank:AM099640] [40]. Unlike the midgut transcriptomes of *P. papatasi *and *L. longipalpis*, we have not found any GSTs of the Delta/Epsilon class, which may be due to the overall low abundance of the GST transcripts in sand fly midguts. Mosquito GSTs play an important role in as antioxidants and knock-down of GSTs of the Theta family has been reported to impact *Plasmodium *infections in *A. gambiae *and *A. stephensi*, although the effect varies with different parasite-vector combinations [41].

A putative peroxiredoxin (or thioredoxin-dependent peroxidase), product of cluster 729, was identified and named *PperPRX *[GenBank:HM119220]. *PperPRX *encodes a putative intracellular protein of 16.7 kDa and a pI of 7.1 containing a peroxiredoxin PRX5-like subfamily domain. Salp25D [GenBank:AF209911], a peroxiredoxin in the tick *Ixodes scapularis*, has been shown to facilitate the acquisition of *Borrelia *from an infected host by detoxifying reactive oxygen species at the vector-pathogen-host interface [42]. Midgut-specific Salp25D, while not significantly aiding the establishment of *Borrelia*, does have a slight protective effect. It is possible that sand fly peroxiredoxins, by detoxifying OH radicals, could have a similar protective effect on *Leishmania *parasites.

### Catalase and superoxide dismutases (SODs)

Transcripts coding for putative enzymes of the superoxide dismutase (SOD)/catalase system were also identified. *PperCat *[GenBank:EZ617712], cluster 852, encodes a putative intracellular protein (molecular weight 57.7 kDa and pI 9.17) containing a catalase domain. It shares high similarity with a putative catalase molecule described in *L. longipalpis *midgut, *LuloCat*, [GenBank:ABV60342] and a similar sequence was also found in the *P. papatasi *midgut cDNA library [GenBank:ES351062]. Catalases are hydrogen peroxide detoxifying enzymes and for an *A. gambiae *homolog [GenBank:AGAP004904], expression is induced in the midgut after blood feeding in response to oxidative stress [43]. In the *P. perniciosus *midgut, the transcript was only found in the sugar fed library (9 sequences) and thus appears to be down regulated by blood feeding. A similar phenomenon could not be observed in *L. longipalpis *due to the low number of catalase sequences found (where one *LuloCat *transcript was found in the blood fed and one in the post-blood fed library infected with *L. chagasi*). The significance of *PperCat *down regulation by blood feeding remains unclear and post-transcriptional regulation cannot be excluded.

**Figure 11:**
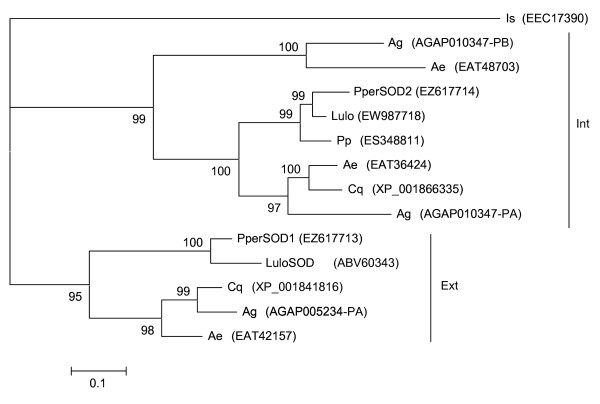
**Phylogenetic analysis of superoxide dismutase molecules from *Ixodes scapularis *(Is), *Anopheles gambiae *(Ag), *Aedes aegypti *(Ae), *Phlebotomus perniciosus *(Pper), *Lutzomyia longipalpis *(Lulo), *Phlebotomus papatasi *(Pp) and *Culex quinquefasciatus *(Cq)**. GenBank accession numbers are given in parentheses, the clades are labelled with the respective localization based on SignalP prediction (Int: intracellular, Ext: extracellular) and node support is indicated by the bootstrap values.

Two clusters with products containing copper-zinc superoxide dismutase (Cu-Zn SOD) domains were identified. *PperSOD1 *[GenBank:EZ617713], cluster 892, encodes a protein similar to a putative secreted Cu-Zn SOD from the midgut of *L. longipalpis*, LuloSOD, [GenBank:ABV60343]. Despite the transcript being 5' truncated, based on homology to the *Lutzomyia *and mosquito molecules we predict the protein possesses a signal peptide and performs a similar function to LuloSOD. The molecule may be secreted or, given that the sequence contains a putative GPI-anchor site, GPI-anchored to the plasma membrane of the midgut cells. *PperSOD2 *[GenBank:EZ617714], cluster 1166, encodes a putative intracellular protein (15.3 kDa, pI 6.3) similar to putative mosquito SODs. Intracellular SODs have not been previously described in the sand fly midgut, but when searched for homologous sequences, we found sequences coding for highly similar proteins in both *L. longipalpis *and *P. papatasi *midgut cDNA libraries [GenBank:EW987718 and GenBank:ES348811, respectively]. Phylogenetic analysis of mosquito and sand fly sequences (Figure 11) shows that extracellular and intracellular Cu-Zn SODs form two distinct clades suggesting the two forms of the enzymes evolved prior to speciation of the two groups of organisms.

### Xanthine dehydrogenase

Cluster 373 [GenBank:EZ617715], *PperXDH*, is a partial transcript coding for a product with high similarity to the C-terminal portion of *L. longipalpis *xanthine dehydrogenase (XDH) [GenBank:CAP08999.1]. XDHs catalyze the oxidation of xanthine to urate, the main product of nitrogen metabolism, which has antioxidant properties in insects. The XDH molecule has been shown to be up regulated after blood feeding in *L. longipalpis*. Silencing of *L. longipalpis *XDH resulted in a reduction in urate production and a reduced life span of both sugar fed and blood fed sand flies [44]. These results suggest that xanthine dehydrogenases are indeed involved in preventing oxidative damage by producing the antioxidant urate in sand flies.

### Ferritin

Two molecules were identified with homology to ferritins described from other insect species including the sand flies *P. papatasi *and *L. longipalpis*. Similar to most insect ferritins, and unlike most vertebrate and plant ferritins, these molecules are likely secreted. *PperFLC *[GenBank:EZ617716], cluster 88, encodes a putative protein with homology to ferritin light-chain subunit and a molecular weight of 24.3 kDa and pI 6.68, once secreted. *PperFHC *[GenBank:EZ617717], cluster 332, encodes a putative ferritin heavy-chain molecule. Although 5' truncated, *PperFHC *has high homology to *P. papatasi *and other species ferritin molecules with signal peptides and is likely similarly secreted. The transcripts originated in similar numbers from the blood fed and sugar fed libraries. Apart from their role in iron metabolism, these sand fly midgut ferritins may also play a role in preventing oxidative damage by sequestering large quantities of free iron from the digested blood meal as was suggested for other blood-feeding insects [45].

### Peritrophic matrix proteins and chitinase

**Figure 12:**
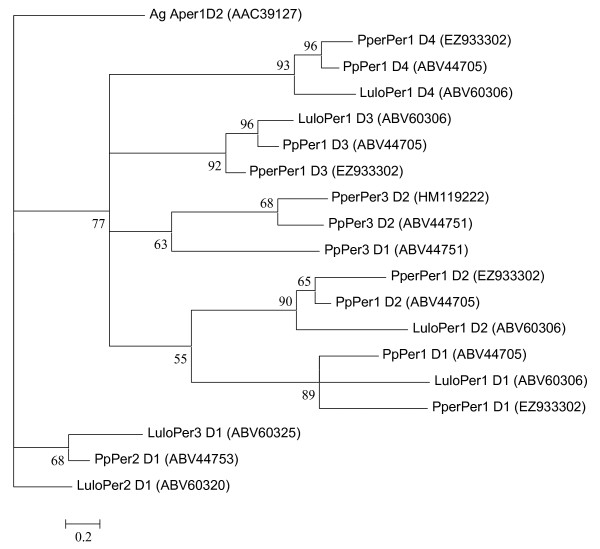
**Phylogenetic analysis of predicted chitin-binding domains of putative peritrophins from *Anopheles gambiae *(Ag), *Phlebotomus perniciosus *(Pper), *Lutzomyia longipalpis *(Lulo) and *Phlebotomus papatasi *(Pp)**. GenBank accession numbers are given in parentheses and bootstrap values indicate node support.

The peritrophic matrix (PM) is an extracellular chitin-containing matrix that is formed in the sand fly midgut after blood feeding that surrounds the ingested blood. Clusters coding for products with similarity to PM proteins described in other blood feeding Dipterans were identified. Three clusters coding for putative peritrophins were detected, originating in higher numbers from the blood fed library. These proteins share homology with molecules previously identified in other sand fly species and contain chitin binding domains (CBDs). *PperPer1 *[GenBank:EZ933302], cluster 97, encodes a protein similar to putative peritrophins with four CBDs previously described from the midgut of *L. longipalpis *(LuloPer1) [GenBank:ABV60306] and *P. papatasi *(PpPer1) [GenBank:ABV44705]. *PperPer1 *is represented by 94 sequences found only in the blood fed library. Although *PperPer1 *ESTs appears to be incomplete at the 5' end and missing the first 13 N-terminal amino acids, based on homology, we predict it to be a secreted molecule of mature molecular weight of 28.2 kDa and pI 4.68. *PperPer2 *[GenBank:HM119221], cluster 330, encodes a putative peritrophin with similarity to a *L. longipalpis *protein LuloPer2 [GenBank:ABV60320]containing one CBD. A third putative peritrophin, *PperPer3 *[GenBank:HM119222], cluster 156, shows similarity to a *P. papatasi *peritrophin PpPer3 [GenBank:ABV44751] and contains two putative CBDs (one partial CBD sequenced due to a 5' truncation). Phylogenetic analysis (Figure 12) of the CBDs from *P. perniciosus*, *P. papatasi *and *L. longipalpis *illustrates a high degree of conservation of the Peritrophin1 arrangement. The four peritrophin domains share respective homology in all the three sand fly species.

A cluster coding for a putative chitinase was identified in the blood fed library. The molecule, named *PperChit *[GenBank:EZ933285] (cluster 124) encodes a putative protein containing a CBD and a mucin-like domain and shares high similarity to *P. papatasi *PpChit1 [GenBank:AAV49322] and *L. longipalpis *LlChit1 [GenBank:AAN71763] chitinases. These midgut-specific, blood meal-induced enzymes have been shown to account for chytinolytic activity in the sand fly midgut and have been implicated in the release of *Leishmania *parasites from the endoperitrophic space [8,46,47].

Along with peritrophins and chitinases, non-chitin binding peritrophic matrix proteins have also been described from bloodsucking insects. Dinglasan et al. [48] performed a proteomic analysis of *A. gambiae *peritrophic matrix and identified a number of non-chitin-binding proteins including proteolytic enzymes and novel classes of PM proteins with unknown function. We identified clusters with homology to these proteins in the *P. perniciosus *libraries. The product of cluster 379 [GenBank:EZ933287] is highly similar to *A. gambiae *PM protein [GenBank:AGAP006398]. The transcript contains a potential N-glycosylation site and 3 DM9 repeats (repeats of unknown function found in a number of arthropod proteins). A homolog was found in the *P. papatasi *midgut library (31.5 kDa midgut protein), [GenBank:ABV44721]. The product of cluster 358 [GenBank:EZ933286] (5' truncated) showed a significant similarity (BLASTp match E = 4e-18) to an *A. gambiae *PM protein [GenBank:AGAP000570]. The sequences originated from both blood fed (3 sequences) and sugar fed (10 sequences) libraries. Homologous proteins were also found in the midgut of *P. papatasi *[GenBank:ABV44744] and *L. longipalpis *[GenBank:ABV60298] and also the salivary glands of *P. duboscqi *[GenBank:ABI20163]. These proteins contain no described conserved domains. Based on homology to the *A. gambiae *protein found in the PM, we speculate that the product of cluster 358 may also be involved in the PM formation in sand flies. On the other hand, its expression in the sugar fed midgut as well as the presence of homologs in the salivary glands may suggest a different function for this protein, such as regulating the haemostatic response.

The structure of the mosquito and sand fly peritrophic matrix is complex and rearranges during the course of blood digestion [49]. The two putative peritrophins with multiple CBDs (PperPer1 and PperPer3) are likely to have a role in cross-linking the chitin fibrils of the peritrophic matrix. In addition to chitin binding, mosquito proteins with CBDs have also been described to bind heme and have a role in its sequestration during blood digestion [50]. Also, the glycosylation of the PM proteins can be of great importance for the PM structure and function. Two of the putative peritrophins, PperPer2 and PperPer3, and the putative chitinase, PperChit, contain mucin-like (Pro-Ser/Thr rich) domains. Glycosylation of these domains can influence the selectiveness of the PM pores, account for water retention within the PM and also protect the molecules from degradation by proteolytic enzymes. Furthermore, the degradation of putatively aglycosylated PM proteins (like PperPer1 and the product of cluster 358) by temporally secreted digestive proteases may play a role in the changes in the PM thickness and structure.

### Transcripts differentially expressed after blood feeding

In order to identify changes in expression of midgut proteins induced by blood feeding, we compared the abundance of transcripts in the sugar fed and the blood fed libraries using chi-square statistical analysis. We found several transcripts that were significantly more abundant after blood feeding and several that were underrepresented in the blood fed library (see tables 3 and 4). As expected, we observed some transcripts putatively involved in blood digestion and peritrophic matrix formation more abundant after blood feeding. These included the putative peritrophin with four chitin-binding domains, *PperPer1*. Similar to the putative orthologues in *P. papatasi *and *L. longipalpis*, *PpPer1 *[GenBank:EU031912] and *LuloPer1 *[GenBank:EU124588], *PperPer1 *was only detected in the blood fed midgut library. With regard to peritrophin sequence abundance, it is interesting to note that we did not detect any peritrophin sequence highly represented before blood feeding. This is in contrast with the situation described in *P. papatasi*, where high numbers of a putative peritrophin with one chitin binding domain, *PpPer2 *[GenBank:EU047543], were detected in sugar fed midguts. In this respect, the observed profile is more similar to peritrophins in the midgut of a more distantly related species *L. longipalpis*.

**Table 3:** ESTs overrepresented in the blood fed library (BF) in comparison to the sugar fed library (SF)

Cluster #	Putative function	SF	BF	P value
45	microvillar protein (PperMVP1)	0	681	7.49E-185
97	peritrophin (PperPer1)	0	94	4.46E-23
81	chymotrypsin (PperChym1)	0	82	2.83E-20
52	microvillar protein (PperMVP5)	0	35	2.10E-09
63	trypsin (PperTryp3)	0	31	1.76E-08
139	microvillar protein (PperMVP4)	0	28	8.67E-08
40	microvillar protein (PperMVP2)	0	26	2.52E-07
79	unknown (lipid recognition)	0	9	2.47E-03
102	chymotrypsin (PperChym2)	2	11	1.14E-02

**Table 4:** ESTs overrepresented in the sugar fed library (SF) in comparison to the blood fed library (BF)

Cluster #	Putative function	SF	BF	P value
46	trypsin (PperTryp1)	513	20	3.16E-115
249	unknown	25	3	3.60E-05
1033	chymotrypsin (PperChym3)	12	0	5.76E-04
16	trypsin (PperTryp2)	10	0	1.68E-03
852	catalase (PperCat)	10	0	2.88E-03
174	glycoside hydrolase (PperGH13)	23	9	1.46E-02
18	microvillar protein (PperMVP3)	18	6	1.56E-02
652	40S ribosomal protein SA	8	1	2.07E-02
183	glycoside hydrolase (PperGH31)	12	3	2.16E-02

Transcripts coding for proteolytic enzymes, namely the chymotrypsins *PperChym1 *and *PperChym2 *and the trypsin *PperTryp3*, were also found more abundant in the blood fed library and thus likely represent digestive enzymes induced by the intake of blood. On the other hand, three other putative proteases, *PperTryp1*, *PperTryp2 *and *PperChym3 *were significantly less abundant in the blood fed library. We speculate that these molecules may be post-transcriptionally regulated digestive enzymes that are stored in the midgut prior to blood feeding.

The most striking differences in sequence abundance before and after blood feeding were observed for the microvillar proteins. Four of the five identified putative microvillar proteins (*PperMVP1*, *2*, *4 *and *5*) were only detected in the blood fed library and in high abundance. This indicates a strong up-regulation of these proteins after the intake of blood. In contrast, *PperMVP3 *was overrepresented in the sugar fed library, suggesting a different role for this protein. The observed microvillar proteins EST distributions are in accordance with what was described in both *P. papatasi *and *L. longipalpis*, where the *PperMVP3 *orthologues, *LuloMVP3 *and *PpMVP3 *were highly represented before blood feeding unlike all other microvillar proteins. The conservation of these proteins and their pattern of expression in the midgut of the three sand fly species indicate their important, yet uncharacterized, roles in the midgut physiology.

The list of sequences overabundant in the blood fed library also includes a putative protein, cluster 79 [GenBank:HQ015441], similar to a putative cockroach allergen MPA2 and several uncharacterized mosquito proteins. The presence of a lipid-binding ML domain in the translated sequence of cluster 79 [Interpro:IPR003172] may suggest a role of lipid recognition. In accordance with their putative function in carbohydrate digestion, putative glycoside hydrolases, cluster 174 [GenBank:HQ015444] and cluster 183 [GenBank:HQ015443], were found overrepresented in the sugar fed library. Interestingly, we also found a putative catalase sequence (PperCat) overabundant in the sugar fed midgut. Similarly, the significance of the higher abundance of a putative 40S ribosomal protein SA (cluster 652) [GenBank:HQ015442] and an unknown, probably non-coding, sequence (cluster 249) [GenBank:GW817178] represented by the clone in the sugar fed midgut, remains unknown.

## Conclusion

*P. perniciosus *is a medically important vector of canine and human visceral leishmaniasis in the Old World. To date, the only molecular data available for this species have been the salivary gland transcripts [51]. This study is the first report on molecules present in the midgut of *P. perniciosus*. As development of *Leishmania *in the vector sand fly is restricted to the digestive tract, the midgut is the primary organ where interactions with *Leishmania *take place. By sequencing and analyzing transcripts present before and after blood feeding, we have provided a catalogue of putative proteins potentially involved in feeding and blood digestion. All the generated ESTs were deposited in the NCBI dbEST database, making them available to scientific communities for further research. Selected molecules of interest were manually annotated and the nucleotide and putative protein sequences submitted to GenBank.

We have identified a variety of molecules, including putative proteins that have not been previously described in the sand fly midgut. Among the putative proteases, these include a putative astacin (PperAstacin2) and a putative chymotrypsin (PperChym5). We also found molecules potentially involved in pathogen recognition such as the gram-negative bacteria binding protein (PperGNBP) and the putative peptidoglycan receptor (PperPGRPLC). Novel putative antioxidant enzymes were also identified including an intracellular superoxide dismutase (PperSOD2) and putative microsomal and Theta class glutathione S-transferases (PperGST2 and PperGST3). In addition, we describe homologs of mosquito peritrophic matrix proteins.

Constructing libraries from sand fly females before and after the intake of blood allowed for the identification of molecules differentially expressed in response to blood feeding. By comparing our findings with the midgut transcriptome analyses of two other sand fly species, *L. longipalpis *and *P. papatasi*, we identified several features shared by the two permissive vectors, *P. perniciosus *and *L. longipalpis*. These include the absence of a significant number of peritrophin sequences before blood feeding, while in *P. papatasi*, a peritrophin with one chitin binding domain was abundant in sugar fed midguts.

Altogether, this study contributes to our knowledge of the molecular background of events that occur in the sand fly midgut. It provides a valuable platform for functional studies of selected molecules relevant in the transmission of *Leishmania*. These may represent targets for use as novel vector-based transmission-blocking vaccines to control this neglected disease.

## Methods

### Sand fly maintenance and dissection

The colony of *Phlebotomus perniciosus *(originally from Spain) was maintained in the insectary of Charles University in Prague as described previously [52]. Adults were kept at 26°C and fed on 50% sucrose ad libitum. Ten midguts from 3-5 days old sugar fed only females were dissected for the sugar fed library construction. Females were fed on an anaesthetised Balb/C mouse and two midguts containing blood were dissected at each of the following time points: 4-6 h, 24 h, 2 days, 3 days and 4 days post-blood meal. These samples were pooled for the construction of the blood fed library. For the qPCR experiment, females were fed through a chick skin on heat-inactivated rabbit blood containing *L. infantum *infected macrophages (or uninfected macrophages in the control group). Midguts from ten infected and ten uninfected (control) females were dissected 6 h, 24 h, 72 h and 10 days after blood feeding and stored individually in 10 μl of RNAlater (Ambion) as well as 10 individual midguts from sand flies before blood feeding (sugar fed). Presence of promastigotes in midguts was confirmed 72 h after blood feeding. On day 10 after blood feeding only sand flies with late-stage infections (with parasites on the stomodeal valve) were used.

### cDNA library construction and sequencing

Messenger RNA was purified from midguts stored in RNAlater (Ambion) using the MicroFastTrack mRNA isolation kit (Invitrogen). PCR-based cDNA libraries were prepared following the instructions for the SMART cDNA library construction kit (Clontech). Each cDNA library was then fractionated into three sets containing small, medium and large fragments using columns provided by the manufacturer. Concentrated cDNA was ligated into a lambda TriplEx2 vector (Clontech). The resulting ligation reaction was packed using the Gigapack III Gold (Stratagene). The libraries thus obtained were plated by infecting log-phase XL1-blue cells (Clontech). Phage plaques lacking β-galactosidase activity were picked using sterile wooden sticks and placed into 75 μl of water. Amplification of the cDNA was performed using Faststart Mix (Roche), 3 μl template and primers PT2F1 (5'-AAG TAC TCT AGC AAT TGT GAG C-3') and PT2R1 (5'-CTC TTC GCT ATT ACG CCA GCT G-3'). The PCR conditions were 1 hold of 75°C for 3 min, 1 hold of 94°C for 4 min, 33 cycles of 94°C for 1 min, 49°C for 1 min, and 72°C for 7 min, 1 hold of 75°C for 7 min. The amplification product was cleaned with three washes with ultra pure water using ExcelaPure plates (EdgeBio) resuspended in 30 μl of water. Sequencing was performed at the Rocky Mountain Laboratories Genomics Unit as described previously [53]. The template was combined with primer PT2F3 (5'- TCT CGG GAA GCG CGC CAT TGT-3') in an ABI 96-well Optical Reaction Plate (P/N 4306737) following the manufacturers recommendations. Sequencing reactions were setup as recommended by Applied Biosystems BigDye Terminator v3.1 Cycle Sequencing Kit by adding 1 * μ*L ABI BigDye Terminator Ready Reaction Mix v3.1 (P/N 4336921), 1.5 * μ*L 5× ABI Sequencing Buffer (P/N 4336699), and 3.5 * μ*L of water for a final volume of 10 * μ*L. Cycle sequencing was performed at 96°C for 10 s, 50°C for 5 s, 60°C for 4 min for 27 cycles on either a Bio-Rad Tetrad 2 (Bio-Rad Laboratories, Hercules, CA) or ABI 9700 (Applied Biosystems, Inc., Foster City, CA) thermal cycler. Fluorescently labelled extension products were purified following Applied Biosystems BigDye XTerminator Purification protocol and then processed on an ABI 3730xL DNA Analyzer (Applied Biosystems, Inc., Foster City, CA).

### Bioinformatic analysis

The bioinformatic analyses of the sequences were performed using the dCAS 1.4 cDNA annotation software [14]. Briefly, primer, vector and low quality sequences were removed at the 5' and 3' ends of each sequence using Cap3 and Phred software [54-56]. Sequences from both libraries were grouped together and aligned to generate clusters based on 95% identity over 100 nucleotides. Three frame translated consensus sequences were supplied to the appropriate BLAST algorithm [57] for comparison to the contents of the NCBI non-redundant protein database, the Gene Ontology database [58], the KOG conserved domain database [59], Simple Modular Architecture Tool (SMART) [60], Protein Family Database (Pfam) [61], rRNA subset database and Mitochondrial and Plasmid Sequences database (MIT-PLA) available from NCBI. The predicted presence of a signal secretion peptide or transmembrane domains was determined using the SignalP and TMHMM programs respectively [62,63]. N- and O-glycosylation site prediction was performed for selected sequences using NetNGlyc 1.0 and NetOGlyc 3.1 software http://www.cbs.dtu.dk/services/NetNGlyc/[64]. Numbers of sequences in the sugar fed and the blood fed library were compared using χ^2 ^statistical analysis. Clusters with significantly unequal distribution of the clone sequences (P < 0.05 and expected frequency in each of the libraries > 4) were identified as over- or underrepresented after blood feeding. Selected sequences were aligned using Clustal × 2.0 [65] and manually refined in BioEdit 7.0 sequence-editing software. For phylogenetic analyses of amino acid sequences, best substitution matrix was determined for each alignment by ProtTest software, version 2.0 [66]. This matrix was then used by TREE-PUZZLE 5.2 [67] to reconstruct maximum likelihood phylogenetic trees from the protein alignments using quartet puzzling with 1000 puzzling steps. Resulting trees were visualized in MEGA 4 [68].

### Macrophage infection

*Leishmania infantum *(MCAN/PT/05/IMT 373) parasites were cultured at 23°C in RPMI medium (Sigma) containing 10% heat-inactivated foetal calf serum (FCS, Gibco), 50 ug/ml gentamicin, 1× BME vitamins (Sigma) and 1% human urine. Mouse macrophage line J774 was cultured at 37°C, 5% CO_2 _in RPMI medium containing 10% FCS, 2 mM alanyl-glutamine and penicillin (200 IU/ml). Macrophages were infected with stationary-phase *L. infantum *parasites at 1:10 macrophage:parasite ratio. After 24 h of co-cultivation at 37°C, 5% CO_2_, non-internalized parasites were removed by washing 3× in the culture media and infected macrophages cultivated overnight. Macrophages were confirmed to contain amastigotes by light microscopy of Giemsa-stained slides. The infected macrophage culture was scraped from the culture plates, centrifuged at 380 g for 10 minutes and resuspended in heat-inactivated rabbit blood for sand fly infections at the concentration of 3 × 10^6 ^macrophages/ml.

### Quantitative PCR

RNA was purified from individual midguts stored in -20°C using a High Pure RNA Tissue Kit (Roche) and cDNA synthesized using SuperScript III Reverse Transcriptase (Invitrogen) with random hexamer primers (Promega) following manufacturer's instructions. Quantification of putative trypsin transcripts was performed by real-time PCR with primers designed for PperTryp1 (5'-CCC AAT GGA CTA TGA CTA CGC-3' and 5'-CGA ACA TCG TCG AAT ACG ATA G-3'), PperTryp2 (5'-GGT GTT CTC GTT GGA GTG GT-3' and 5'-TGG CGT AAA CTC CAG GGT AG-3'), PperTryp3 (5'-TGA GGA TGT TGA GGA TGG AA-3' and 5'-CTC TTG GTT ATT CAG AGT GTT ACC C-3') and PperS7 ribosomal protein as a reference transcript (5'-ATC CCT ATG CCG AAG CAG A-3' and 5'-TCA AGC TCA CGT ACC AGA CG-3'). The amplification reaction was carried out using the iQ5 real-time PCR detection system (Bio-Rad) by using the SYBR Green detection method (iQ SYBR Green Supermix, Bio-Rad) in 15 μl reaction volume containing 1 μl cDNA template and 0.5 μl primer set (5 uM each). The running conditions were as follows: 3 min at 95°C followed by 40 repetitive cycles: 10 s at 95°C, 10 s at 55°C, and 10 s at 72°C. Reactions were run in duplicates and data were analysed using the 2^-ΔΔCT ^(Livak) method. In a preliminary experiment we have established that the target genes (trypsins) and the reference gene (PperS7 ribosomal protein) have similar (about 5% variance) amplification efficiencies which are nearly 100%. Data are presented as relative transcript levels using the S7 ribosomal protein gene as an internal control. Statistical analysis was done by Mann-Whitney U Test using (Statistica 6.1 StatSoft software).

## Authors' contributions

AD participated in the study design, sand fly rearing, dissections, cDNA library construction and annotation, sequence alignment, phylogenetic analysis, quantitative PCR and drafting the manuscript. AJF and KDB sequenced all cDNA amplification products selected from the library. JV participated in the quantitative PCR experiment. PV and JGV participated in the study design and coordination, and revised the manuscript. RCJ conceived the study, participated in its design and coordination and revised the manuscript. All authors read and approved the final manuscript.
